# SERPINE2, an inhibitor of plasminogen activators, is highly expressed in the human endometrium during the secretory phase

**DOI:** 10.1186/1477-7827-9-38

**Published:** 2011-03-23

**Authors:** Robert Kuo-Kuang Lee, Chi-Chen Fan, Yuh-Ming Hwu, Chung-Hao Lu, Ming-Huei Lin, Ying-Jie Chen, Sheng-Hsiang Li

**Affiliations:** 1 Department of Medical Research, Mackay Memorial Hospital, Taipei, Taiwan; 2 Department of Obstetrics and Gynecology, Mackay Memorial Hospital, Taipei, Taiwan; 3 Department of Obstetrics and Gynecology, Taipei Medical University, Taipei, Taiwan; 4 Department of Physiology, Mackay Memorial Hospital, Taipei, Taiwan; 5 Department of Medical Laboratory Science and Biotechnology, Yuanpei University, Hsinchu, Taiwan; 6 Mackay Medicine, Nursing and Management College, Taipei, Taiwan; 7 Graduate Institute of Biotechnology, National Taipei University of Technology, Taipei, Taiwan

## Abstract

**Background:**

SERPINE2, also known as protease nexin-1, belongs to the serine protease inhibitor (SERPIN) superfamily. It is one of the potent SERPINs that modulates the activity of plasminogen activators (PAs). PAs and their SERPIN inhibitors, such as SERPINB2 and SERPINE1, were expressed in the human endometrium and were implicated in implantation. However, expression data about SERPINE2 in the human endometrium is still unknown. Thus, we conducted an investigation to reveal the spatiotemporal and cellular expression of SERPINE2 in the human uterus during the menstrual cycle.

**Methods:**

Seven patients who underwent a hysterectomy and samples of 120 archived patients' endometrial curettage or parts of the uterus that were formalin-fixed and embedded in paraffin. Western blotting was performed to evaluate the specificity and sensitivity of the antibody. Immunohistochemistry was conducted to localize the SERPINE2 expression site. Quantitative analysis was conducted to evaluate expression levels of SERPINE2 in various sub-phases of the menstrual cycle.

**Results:**

The SERPINE2 protein was primarily detected in the uterine fluid during the mid- and late-secretory phases of the menstrual cycle. It was predominantly expressed in the luminal and glandular epithelium, less in the myometrium, and only dispersedly in certain stromal cells throughout the menstrual cycle. A quantitative analysis of expression levels of SERPINE2 in the glandular epithelium revealed that it was highly expressed in the endometrium during the secretory phase compared to the proliferative phase.

**Conclusions:**

The SERPINE2 protein is highly expressed in the endometrium during the secretory phase, indicating that it may participate in tissue remodeling involved in implantation.

## Background

The plasminogen activator (PA) system refers to the PA and its cognate inhibitors [1]. The system includes two forms of PA, tissue-type (PLAT or tPA) and urokinase-type (PLAU or uPA), and four forms of serine protease inhibitors (SERPIN), including SERPINA5 (also called protein C inhibitor, PCI), SERPINB2 (also called plasminogen activator inhibitor 2, PAI-2), SERPINE1 (also called plasminogen activator inhibitor 1, PAI-1), and SERPINE2 (also called protease nexin-1, PN-1) [2].

The PA system is associated with many reproductive processes, including ovulation, embryogenesis, and embryo implantation in female reproductive tissues [1,3]. How SERPIN modulates the proteolytic activities of PLAT/PLAU in reproductive tissue remodeling is of great importance.

Tissue remodeling requires a fine-tuned balance between levels of proteases and their cognate inhibitors. The PA is involved in tissue remodeling by converting abundant extracellular plasminogen into plasmin, an active protease, which degrades the extracellular matrix. The classical substrate of plasmin is fibrin, but in fact, many other matrix proteins can be cleaved by this enzyme [1].

The expression and activity of PLAT and PLAU were detected in the human uterus, including the uterine fluid [4-6], and the endometrium during cycling [5-9] and implantation [10]. SERPINB2 [8] and SERPINE1 [7,8] were demonstrated to be present in the human endometrium. SERPINE1 was even detected in human and mouse uteri during implantation [10,11], indicating that the PA inhibitor is involved in implantation.

SERPINE2 has broad anti-protease activity specific to serine proteases, including trypsin, thrombin, plasmin, PLAU [12], and prostasin [13]. It is widely expressed in various tissues [14]. In the uterus, it is reported that expression levels of SERPINE2 in the monkey endometrium and placenta during early pregnancy were weak or below the level of detection [15]. In rats, *Serpine2 *messenger (m)RNA was exclusively detected in endometrial stromal cells of the uterus, in particular on day 6.5 postcoitally, thus suggesting that it may be involved in the implantation process [16].

Recently, SERPINE2 protein was reported to be expressed in the murine uterus during the estrous cycle, pregnancy, and lactation [17]. It is predominantly expressed in the luminal and glandular epithelium and weakly in stromal cells and myometrium. It seems that different species have different expression patterns for the SERPINE2 protein. So far, there is no study on this aspect in the human uterus. Herein, we conducted an investigation to reveal the spatiotemporal and cellular expression of SERPINE2 in the human uterus during the menstrual cycle.

## Methods

### Sample collection

Uterine fluid aspirates were collected under the consent of patients (n = 7) who were to undergo a hysterectomy because of a leiomyoma or adenomyosis. After anesthesia and before surgery, uterine fluid in the cavity was directly aspirated using an embryo transfer catheter (Labotect, Goettingen, Germany). Then, the cavity was flushed using 3 mL of saline. The two solutions were mixed for analysis. On day 1 of the operation, blood was aspirated to examine the serum concentration of estradiol and progesterone to evaluate the phase of the menstrual cycle. A sample from endometrial curettage was also formalin-fixed and paraffin-embedded (FFPE) for a histological evaluation. Menstrual cycle can be dated into 6 sub-phases according to the anatomical changes within the endometrial biopsy including, early proliferative (EP) (days 5-7), mid-proliferative (MP) (days 8-10), late proliferative (LP) (days 11-14), early secretory (LS) (days 14-21), mid-secretory (MS) (days 22-24), and late secretory (LS) (days 25-28) phases [18]. Informed consent for all samples was obtained from all patients.

To analyze the SERPINE2 protein expression in different phases of the menstrual cycle, 120 archived FFPE samples of endometrial curettage or parts of the uterus from patients of reproductive age at various sub-phases of the menstrual cycle judged by a pathologist were obtained from the Department of Pathology, Mackay Memorial Hospital (Taipei, Taiwan). Wax blocks, 25 cases of EP, 20 cases of MP, 14 cases of LP, 20 cases of ES, 21 cases of MS, and 20 cases of LS, were prepared from patients who were to undergo a hysterectomy because of a leiomyoma or adenomyosis. All retrieved wax blocks had been treated at the hospital for 2 years. The Institutional Review Board approved this study (reference number: MMH-I-S-441).

### Western blotting

To assess the specificity of the antibody, 400 ng of recombinant glutathione S-transferase (GST)-human SERPINE2 protein (Abnova, Taipei, Taiwan) was resolved by sodium dodecylsulfate polyacrylamide gel electrophoresis (SDS-PAGE) on a 10% gel slab (8.2 × 7.3 × 0.075 cm) and was transferred to a nitrocellulose membrane for immunostaining according to a previously described method [19]. Membranes were blocked with 10% (w/v) skim milk in phosphate-buffered saline (PBS) (blocking solution) for 2 h, and then incubated with our homemade anti-mouse SERPINE2 antiserum (1: 5000) [17,20], anti-human SERPINE2 antibody (1: 1000, catalog no. AF2980, R&D Systems, Minneapolis, MN, USA), or another anti-human SERPINE2 antibody (1: 1000, product no. H00005270-B01, Abnova) in blocking solution for 2 h at 37°C. After gentle agitation in four changes of PBS for 15 min each, membranes were immunoreacted with horseradish peroxidase (HRP)-conjugated goat anti-rabbit immunoglobulin G (IgG) (1: 5000, GE Healthcare Life Sciences, Piscataway, NJ, USA), donkey anti-goat IgG (1: 3000, Jackson ImmunoResearch, West Grove, PA, USA), or horse anti-mouse IgG (1:3000, Cell Signaling Technology, Beverly, MA, USA), respectively, in blocking solution for 2 h at 37°C. After gentle agitation as mentioned above, immunoreactive bands were revealed using an enhanced chemiluminescent substrate according to the manufacturer's instructions (Western Lightning; PerkinElmer, Boston, MA, USA).

To evaluate the sensitivity of the antibody, 100 μg of a tissue extract from endometrial curetting was resolved, transferred onto a blot, and detected using anti-mouse SERPINE2 antiserum (1: 5000) or an anti-human SERPINE2 antibody (1: 1000) following the method described above.

To examine the expression of SERPINE2 in the uterine fluid, 50 μg of uterine luminal proteins was applied and detected using anti-mouse SERPINE2 antiserum (1: 3000) following the same method.

### Immunohistochemical staining and signal analysis

Immunohistochemical analysis was performed on an automatic staining machine (BenchMark XT, Ventana Medical Systems, Tucson, AZ, USA) using the iVIEW 3, 3-diaminobenzidine (DAB) detection kit (Ventana Medical Systems). After tissue sections (4 μm) on slides were deparaffinized and hydrated, they were heated to 95°C for 8 min and then 100°C for 4 min to induce antigen retrieval using Ventana Cell Conditioner 1 (Ventana Medical Systems). After cooling to room temperature for 30 min, the slides were treated with an iVIEW inhibitor at 37°C for 4 min to inactivate the endogenous peroxidase activity. Slides were then incubated with anti-SERPINE2 antiserum or antiserum pretreated with SERPINE2 antigen-conjugated beads diluted 1: 1000 in blocking solution at 37°C for 16 min. After rinsing with PBS, slides were treated with iVIEW biotin-conjugated IgG in blocking solution for 8 min at room temperature. Slides were rinsed again and then incubated with iVIEW streptavidin-conjugated HRP in blocking solution for 8 min at room temperature. Protein signals were developed by iVIEW DAB and hydrogen peroxide for 8 min at 37°C. Slides were finally incubated with iVIEW copper for 4 min to enhance the signal intensity, counterstained with hematoxylin (Vector Laboratories, Burlingame, CA), and photographed with a Nikon Eclipse E600 microscope (Tokyo, Japan) or Zeiss AxioImager Z1 microscope system (Wetzlar, Germany) equipped with a CCD camera and an automated acquisition system (TissueGnostics, Vienna, Austria). Pictures were acquired using TissueFaxs software (TissueGnostics). The percentage of positively stained cells was determined using HistoQuest software (TissueGnostics). Five endometrial glands on a slide for each individual patient at a given sub-phase of the menstrual cycle were chosen to evaluate the level of staining according to the mean intensity of DAB, refers to the staining signal of SERPINE2, of the stained cells which were counterstained with hematoxylin. The strong intensity was suggested that more than 50% of glandular epithelial cells had a staining intensity of > 15. The weak intensity was suggested that more than 60% of glandular epithelial cells had a staining signal of < 10. The staining signal other than the two cases was referred to the medium signal. A chi-square test was performed to compare the significance of difference in expression levels of SERPINE2 among patients at different sub-phases of menstrual cycle.

## Results

**Figure 1:**
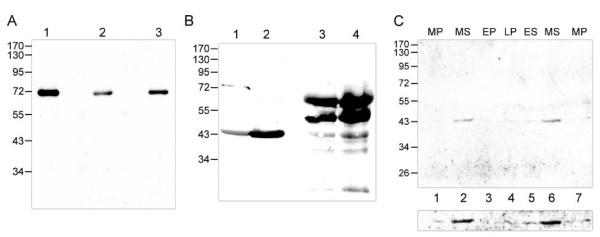
**Antibody specificity and presence of the SERPINE2 protein in human uterine fluid**. (A) Four hundred nanograms of recombinant human SERPINE2 was resolved on 10% SDS-PAGE and followed by Western blotting using anti-mouse SERPINE2 antiserum (lane 1), an anti-human SERPINE2 antibody (R&D) (lane2), or another anti-human SERPINE2 antibody (Abnova) (lane 3). (B) One hundred micrograms of the extract of endometrial curettage was analyzed by anti-mouse SERPINE2 antiserum (lanes 1 and 2) and an anti-human SERPINE2 antibody (R&D) (lanes 3 and 4). (C) Fifty micrograms of uterine fluid proteins collected from each individual patient (*n *= 7) was Western-blotted using anti-mouse SERPINE2 antiserum (1:3000) (upper panel). EP, MP, and LP indicate early-, mid-, and late-proliferative phases, and ES and MS indicate early- and mid-secretory phases, respectively. The blot was also overexposed to clearly display the staining signal (lower panel).

To assess the specificity of the antibodies, we analyzed two commercially available anti-human SERPINE2 antibodies and homemade anti-mouse SERPINE2 antiserum by Western blotting. As shown in Figure 1, all antibodies could recognize the recombinant GST-human SERPINE2 protein (Figure 1A), indicating that our antibody has specificity for the human SERPINE2 protein.

To evaluate the sensitivity of the antibody, a tissue extract from endometrial curettage was resolved on a gel and Western-blotted using the three antibodies. An apparent 44-kDa protein band corresponding to human SERPINE2 was immunodetected by our anti-mouse SERPINE2 antibody, while many non-specific crossed-reacted protein bands were blotted by the commercial antibodies. In addition, an approximately 75-kDa protein band corresponding to the complex of SERPINE2 and a certain protease demonstrated in previous studies [14,17,21,22] was found. Thus, the sensitivity of our antibody apparently excelled those of the commercial antibodies (Figure 1B). These data indicated that our antibody has high specificity and sensitivity for detecting the human SERPINE2 protein.

Using the antibody, SERPINE2 was detected in the endometrial fluid of the human uterus (Figure 1C). It was obvious that there was more SERPINE2 in the secretory phase (lanes 2 and 6) than in the proliferative phase (lanes 1, 3, 4, and 7). Thus, SERPINE2 is indeed a human uterine secretory protein.

**Figure 2:**
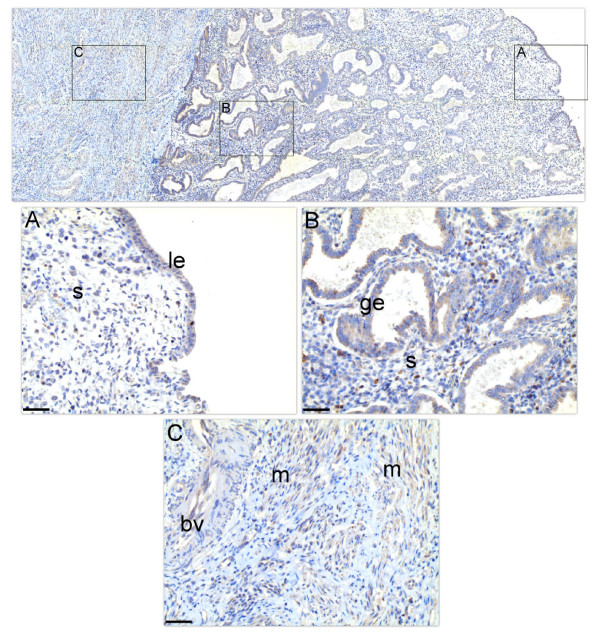
**Localization of SERPINE2 in the human uterus**. Longitudinal sections of the early secretory phase uterus (n = 5) on the slide were incubated with anti-mouse SERPINE2 antiserum and then treated with biotin-conjugated goat-anti-rabbit IgG and HRP-conjugated streptavidin (brown). For contrast, specimens were further stained with hematoxylin (blue). The representative picture is shown. Magnified pictures of the luminal epithelium (A), glandular epithelium (B), and myometrium (C) are shown. Bar = 50 μm. bv, blood vessel; ge, glandular epithelium; le, luminal epithelium; m, muscle; s, stroma.

An immunolocalization study was conducted to reveal the cellular localization of the SERPINE2 protein in the early secretory phase uterus. As shown in Figure 2, SERPINE2 was primarily detected on the apical surface of the luminal epithelium (Figure 2A) and glandular epithelium (Figure 2B), but weakly on the myometrium (Figure 2C). In contrast, the protein signal in stromal cells was dispersedly distributed on some unidentified cells, parts of which may be macrophage as judged by cell morphology (Figure 2B). In addition, SERPINE2 was detected on endothelial cells of the vessel (Figure 2C) as demonstrated by a previous study [23]. When slides were immunostained with control antiserum, no signal was detected (data not shown).

**Figure 3:**
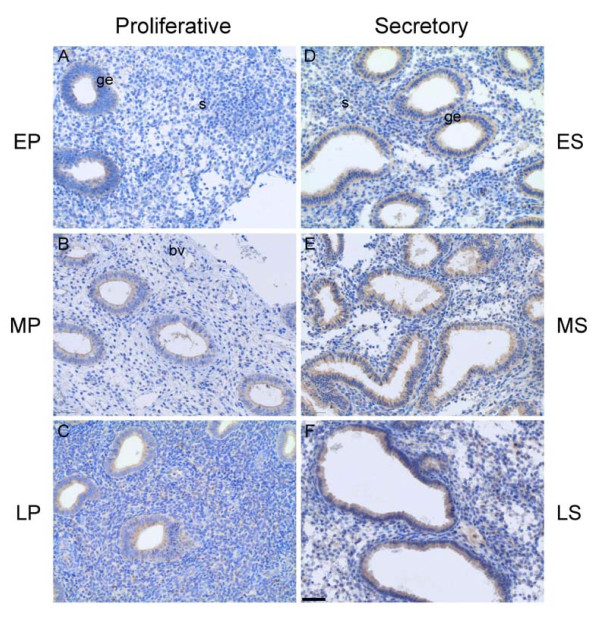
**Glandular epithelial expression of the SERPINE2 protein in the endometrium during the menstrual cycle**. Sections prepared from endometrial curettage during early-proliferative (EP), mid-proliferative (MP), late-proliferative (LP), early-secretory (ES), mid-secretory (MS), and late-secretory (LS) phases were incubated with anti-mouse SERPINE2 antiserum and then treated as described in Figure 2. Bar = 50 μm. bv, blood vessel; ge, glandular epithelium; s, stroma.

To further evaluate the expression of the SERPINE2 protein in various sub-phases of the menstrual cycle, we examined endometrial slides prepared from early-, mid-, and late-proliferative as well as secretory phases by immunohistochemistry. The results showed that the signal was relatively weaker during the proliferative phase (Figure 3A-C), while it was strongly detected in the glandular epithelium during the secretory phase (Figure 3D-F), especially in the mid- and late-secretory phase (Figure 3E, F).

**Figure 4:**
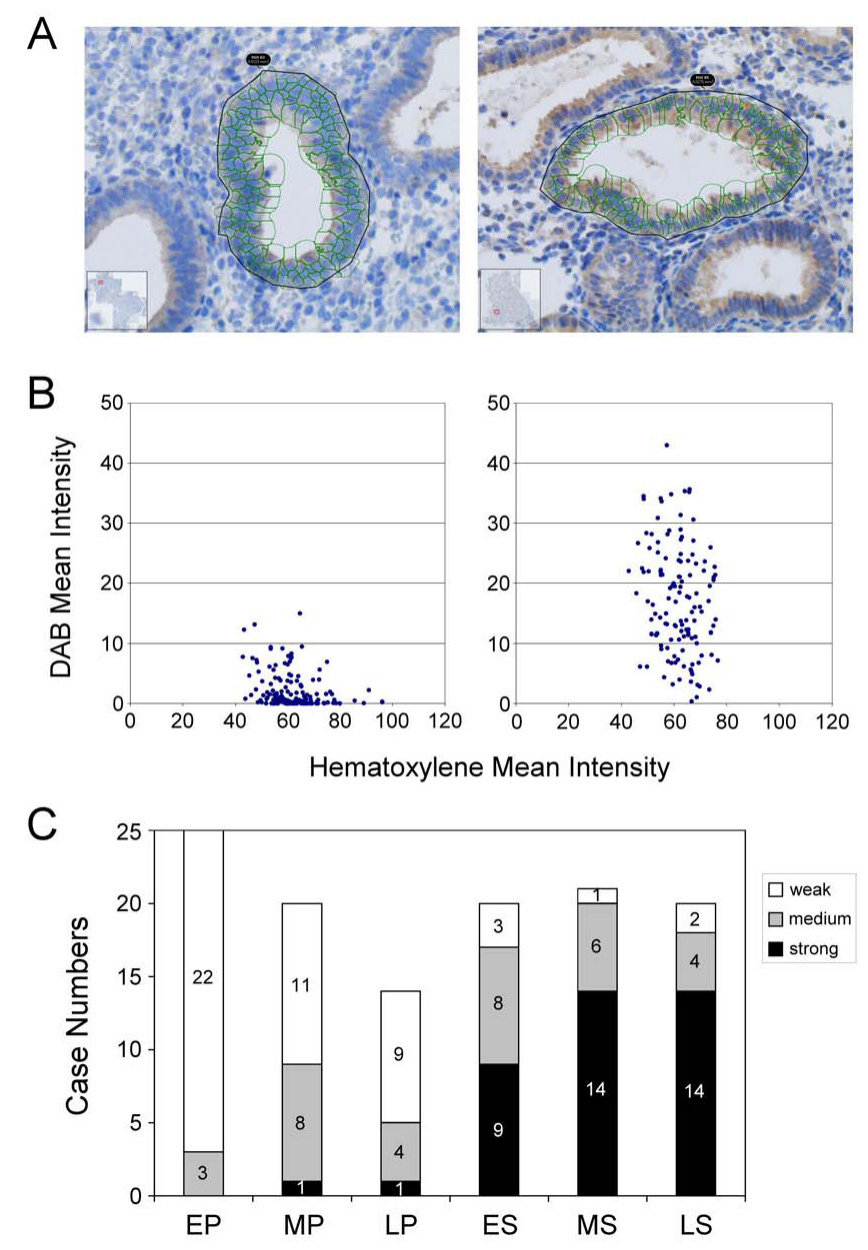
**Quantification of SERPINE2 protein expression levels in endometrial glands**. Representative samples were analyzed by automated cell acquisition and quantification software (A). The expression signal of a respective glandular gland was quantified using HistoQuest software and is presented as a scattergram. Each spot on the scattergram stands for the intensity of one cell (B). The relative SERPINE2 protein expression levels in patients' glandular glands at various sub-phases of the menstrual cycle are shown as bar diagrams (C). Differences are significant among patients at various groups (χ = 69.32, *p *< 0.0001).

Positively stained cells in the endometrial gland were quantified to analyze expression levels of the SERPINE2 protein. The signal intensity was determined by quantitative software. Scattergrams of the staining intensity (Figure 4B) indicated strongly and weakly stained cells in the glandular epithelium (Figure 4A). The results implied that weakly stained cells in the gland were mostly derived from the proliferative phase, while strongly stained cells were predominantly from the secretory phase (Figure 4C). Thus, SERPINE2 was primarily expressed in secretory-phase endometrial glandular cells from which it was secreted into the lumen of the uterus.

## Discussion

In this study, we demonstrated that the SERPINE2 protein, an inhibitor of PAs, is highly expressed in the human uterus during the secretory phase. However, its levels are low in the uterus during the proliferative phase. It is primarily expressed in the luminal and glandular epithelium, weakly expressed in the myometrium, and dispersedly expressed by certain stromal cells.

Proteases are known to be involved in extracellular matrix degradation required for implantation, including cysteine, serine, and matrix metalloproteinases [24]. PA serine proteases and their cognate inhibitors are involved in implantation [1,3]. Tissue remodeling is an important biological event for many reproductive processes that occur in the ovary, uterus, and placenta, such as follicle growth, ovulation, the estrous cycle, implantation, and placentation [1,3]. SERPINE2 was previously demonstrated to primarily be upregulated in dominant follicles during follicle growth in cattle [25,26] and during ovulation in mice [27]. It is also highly expressed in the rodent uterus during implantation and pregnancy [17].

Similar to the rodent data, in this study, we revealed that the SERPINE2 protein was highly expressed in the glandular epithelium of human uterus around the time of the implantation window. These data suggest a role for SERPINE2 in regulating tissue remodeling during implantation.

Human SERPINE2 has high amino-acid identities with the rat and mouse SERPINE2, at 100% and 89%, respectively. Anti-mouse SERPINE2 antiserum can cross-react with rat SERPINE2 in the uterus [17], indicating that it would cross-react with human SERPINE2. In this study, we demonstrated that anti-mouse SERPINE2 antiserum recognized the human SERPINE2 protein. Interestingly, we found that only one SERPINE2 protein form existed in the human uterus, while two isoforms were found in the mouse and rat uterus [17].

Our anti-SERPINE2 antibody, produced using a highly purified protein as the immunogen [17], was very sensitive at detecting tissues in which there was trace expression of SERPINE2 [17,20], even better than the commercial antibodies. In this study, the commercial antibodies could not specifically detect SERPINE2's expression in endometrial tissues, demonstrating the sensitivity of our antibody. Weak or very low levels of SERPINE2 in the monkey endometrium and placenta in a previous study may have resulted from the fact that they used the peptide or *Escherichia coli*-expressed protein as the immunogen which is often not in the native conformation of the protein [15].

Previous studies demonstrated that SERPINE2 can form a complex of approximately 75 kDa with PLAU [14,21,22] or about 82 kDa with prostasin [13]. A 75-kDa protein complex was found in the protein extract of murine and rat uteri [17]. Similarly, the complex was also detected in a human endometrial tissue extract (Figure 1B), indicating that SERPINE2 may act in the human uterus with a certain protease that may be involved in tissue remodeling.

Prostasin is highly expressed, but its inhibitor, SERPINE2, is barely expressed in the glandular epithelium of the monkey endometrium during early pregnancy [15]. However, the SERPINE2 protein is highly expressed in the human endometrium, although prostasin protein expression in the human uterus is still unknown. Whether SERPINE2 regulates the proteolytic activity of prostasin in the human uterus awaits further investigation.

PLAT, PLAU, SERPINE1, and SERPINB2 were found to be expressed in the human endometrium [5-10]. PLAU mRNA is expressed by stromal cells in all phases of the menstrual cycle. However, the PLAU protein is expressed by epithelial and stromal cells in the early-proliferative and late-secretory phases but is nearly undetectable in the mid-secretory phase of the menstrual cycle [7]. SERPINE1 mRNA and protein were reported to predominantly be expressed by stromal cells in the late secretory phase [7]. The SERPINB2 protein was found at very low concentrations and was expressed by certain stromal cells in the human endometrium. Expression levels of this protein showed no difference between the proliferative and secretory phases [8]. Our preliminary results in analyzing the relative mRNA expression levels of PA inhibitors showed that *SERPINE2 *mRNA was the most highly expressed PA inhibitor in the extract of the human uterus (unpublished observations). Thus, the SERPINE2 protein may be the major PA inhibitor in the human uterus. This was also found in the mouse uterus [17].

While SERPINE2 was expressed in the mouse and rat uteri [17], it was below the level of detection in the monkey uterus [15]. This is the first report to demonstrate that SERPINE2 is prominently expressed by the human endometrium. We also found that the SERPINE2 protein is present in the uterine fluid. PA activity was also demonstrated to be present in the uterine fluid [4-6]. Thus, the SERPINE2 protein may exert an inhibitory role of modulating PA activity in the uterine milieu.

In conclusion, cellular localization of the SERPINE2 protein in the human uterus suggests that it may play important roles in PA-modulated tissue remodeling. The high expression of the SERPINE2 protein in the secretory phase suggests that it might be associated with embryo implantation.

## Competing interests

The authors declare that they have no competing interests.

## Authors' contributions

RKKL participated in the design of the study and helped draft the manuscript. CCF carried out immunohistochemistry and drafted parts of the manuscript. YMH and MHL participated in sample collection. CHL carried out Western blotting. YJC helped the immunohistochemical signal quantification. SHL conceived of the study, and participated in the project design and coordination. All authors read and approved the final manuscript.
